# Oxidative Damage and Energy Metabolism Disorder Contribute to the Hemolytic Effect of Amorphous Silica Nanoparticles

**DOI:** 10.1186/s11671-016-1280-5

**Published:** 2016-02-02

**Authors:** Lizhen Jiang, Yongbo Yu, Yang Li, Yang Yu, Junchao Duan, Yang Zou, Qiuling Li, Zhiwei Sun

**Affiliations:** School of Public Health, Capital Medical University, Beijing, 100069 People’s Republic of China; Beijing Key Laboratory of Environmental Toxicology, Capital Medical University, Beijing, 100069 People’s Republic of China

**Keywords:** Amorphous silica nanoparticles, Hemolytic effects, Human erythrocytes, Oxidative damage, ATPase activity

## Abstract

Amorphous silica nanoparticles (SiNPs) have been extensively used in biomedical applications due to their particular characteristics. The increased environmental and iatrogenic exposure of SiNPs gained great concerns on the biocompatibility and hematotoxicity of SiNPs. However, the studies on the hemolytic effects of amorphous SiNPs in human erythrocytes are still limited. In this study, amorphous SiNPs with 58 nm were selected and incubated with human erythrocytes for different times (30 min and 2 h) at various concentrations (0, 10, 20, 50, and 100 μg/mL). SiNPs induced a dose-dependent increase in percent hemolysis and significantly increased the malondialdehyde (MDA) content and decreased the superoxide dismutase (SOD) activity, leading to oxidative damage in erythrocytes. Hydroxyl radical (·OH) levels were detected by electron spin resonance (ESR), and the decreased elimination rates of ·OH showed SiNPs induced low antioxidant ability in human erythrocytes. Na^+^-K^+^ ATPase activity and Ca^2+^-Mg^2+^ ATPase activity were found remarkably inhibited after SiNP treatment, possibly causing energy sufficient in erythrocytes. Percent hemolysis of SiNPs was significantly decreased in the presence of *N*-acetyl-cysteine (NAC) and adenosine diphosphate (ADP). It was concluded that amorphous SiNPs caused dose-dependent hemolytic effects in human erythrocytes. Oxidative damage and energy metabolism disorder contributed to the hemolytic effects of SiNPs in vitro.

## Background

Nanotechnology has been rapidly developed as a pioneer for advanced science in the fields of biology and medicine [1]. Nanomaterials are providing remarkable advantages in medical applications via the efficient absorption of diverse molecules, including minor drugs, gene vector, antibiotics, and antibodies [2–5]. Among the top five widely used engineering nanomaterials, silica nanoparticles (SiNPs) are extensively applied in bioimaging, gene therapy, and drug delivery [6]. Compared with traditional drugs, the SiNP-based drugs changed pharmacokinetics by increasing solubility and availability [7]. The benefits of SiNPs in biomedical applications also brought concerns about the safety of SiNPs, especially in evaluation of blood compatibility and stability since intravenous injection becomes a common iatrogenic exposure route of SiNPs and SiNPs could directly interact with the blood components [7, 8].

Erythrocytes are the dominant cell type (90 %) in blood, and the hemolytic effects of nanoparticles via direct interaction with erythrocytes have been gaining growing attentions. Silver nanoparticles (AgNPs) induced size-dependent adsorption, uptake, and hemolytic activity in *Carassius* red blood cells (RBCs) [9]. The endocytosis of mesoporous silica nanoparticles (MSNs) was clearly observed in various cell lines [10], and a size- and surface-dependent interaction of MSN with RBCs has demonstrated a strong affinity and altering membrane shape ability of MSN [11]. However, studies based on hemolysis assays showed that MSNs (MCM-41-type) were innocuous compared to amorphous SiNPs [12]. The hemolytic effects on RBCs were confirmed to be related to nanoparticle porosity, geometry, and surface functionality [13]. Although quite a few types of nanomaterials such as mesoporous and nonporous SiNPs [11, 13], TiO_2_ nanoparticles [14], and polymer nanoparticles [15] were demonstrated to induce the hemolytic effects in blood circulation, the research of biocompatibility and bioavailability of SiNPs still cannot match their rapid pace of invention and application in new forms.

Erythrocyte species were proven to be related to the hemolysis of nanoparticles. Diesel exhaust particles (DEP), as the main component of PM_2.5_ and ultrafine particles, were reported to induce slight hemolytic effects on human erythrocytes (0.7 %) and mouse erythrocytes (2.5 %) but remarkably on rat erythrocytes in a dose-dependent manner [16]. TiO_2_ nanoparticles had the direct hemolytic effect in rabbit erythrocytes [14], and AgNPs showed prominent hemolytic toxicity in human blood [17]. Nemmar et al. [18] have proven that amorphous SiNPs could cause hemolytic effects in mouse erythrocytes, but it still remains unclear whether amorphous SiNPs induce hemolysis effects in human erythrocytes. Therefore, human RBCs were selected to fully understand the hemocompatibility of amorphous SiNPs.

Previously, we have noticed that intravenous administration of amorphous SiNPs decreased the amount of RBCs and content of hemoglobin in rats. Moreover, the occult blood tests and bilirubin in urine were shown positive (unpublished observations). All these results suggested that hemolytic anemia might generate in vivo after SiNP exposure. In in vitro studies, human platelets were decreased and aggregated by the SiNPs, mostly due to the consumption and destruction of platelets via interaction with SiNPs [19, 20]. These findings illustrated the urgent need for investigations on the hemolytic effects of SiNPs in erythrocytes in vitro and the potential mechanisms of erythrocyte rupture. Many researches provided evidences that oxidative damage contributed to the adverse effects of SiNPs [21, 22]. In our previous studies, we have clarified that oxidative stress mediated by reactive oxygen (ROS) generation could induce cell membrane injuries, organelle damage, and cell death [23, 24]. We also demonstrated that SiNPs induced a decrease in mitochondrial membrane potential (MMP) and resulted in cell energy metabolism disorder, which contributed to apoptotic cell death in endothelial cells [25]. As a result, we hypothesized that oxidative stress and adenosine triphosphate (ATP) metabolism disorder were conducive to the erythrocyte membrane rupture, leading to hemolysis.

As well known, blood is a complex fluid with abundant proteins, ions, and blood cells. The changes on aggregation and dispersion stability of nanoparticles would be induced by forming “protein coronas” when nanoparticles directly interacted with serum proteins [26]. The surface property alteration based on blood ionic strength can also induce nanoparticle agglomerations, which further retard cell motility and functions [27]. Fedeli et al. [28] found that albumin ratio in serum determined the distinct hemolytic effects of SiNPs on human erythrocytes, suggesting serum protein coronas played a role in promoting hemolysis. However, there are quite a few studies that chose the protein-free media to create a controllable condition to focus on the interaction between the nanoparticles and erythrocytes [11, 29, 30]. In the present study, human erythrocytes were incubated with amorphous SiNPs in the serum-free media (phosphate-buffered saline (PBS)), and the hemolytic effects and morphology of erythrocytes were detected. The hydroxyl radical (·OH) levels were measured by electron spin resonance (ESR) to measure ROS accumulations in erythrocytes. The malondialdehyde (MDA) content and superoxide dismutase (SOD) activity were further measured to determine the oxidative damage of erythrocyte membrane caused by SiNPs. The ROS scavenger *N*-acetyl-cysteine (NAC) was selected to elucidate the relationship between ROS and hemolytic toxicity of amorphous SiNPs. ATPase activities of erythrocyte membrane and the protective effect of adenosine diphosphate (ADP) in hemolysis were measured to find out if energy shortage is the potential explanation for hemolysis induced by amorphous SiNPs.

## Methods

### Amorphous Silica Nanoparticle Preparation

Amorphous SiNPs were synthesized using the Stöber method by the School of Chemistry, Jilin University [22]. The morphology characteristics of SiNPs were determined by transmission electron microscope (TEM) (JEOL, Japan), and Image J software was used to detect the size distribution. A zeta electric potential granulometer (Malvern Nano-ZS90, UK) was employed to measure the zeta potential and hydrodynamic sizes of SiNPs in PBS. The purity of SiNPs was evaluated by ICP-OES (Thermo Fisher Scientific, Switzerland). The endotoxins of SiNPs were detected by gel-clot limulus amebocyte lysate (LAL) assay.

### Blood Collection

Sodium citrate-stabilized human blood samples were freshly collected from the Clinical Examination Center of Beijing Xuanwu Hospital, and the project was approved by the Ethics Committee at Capital Medical University. Whole blood was centrifuged at 3500 rpm for 5 min, and packed erythrocytes were isolated from the plasma. The packed RBCs were washed three times with PBS (pH 7.4), and after cell washing, 100 μL of packed RBCs was diluted to 0.9 mL of PBS to prepare the RBC suspensions.

### Hemolysis Assay Incubated with SiNPs

An amount of 0.2 mL of diluted RBC suspensions was separately mixed with 0.8 mL of amorphous SiNP suspensions at the concentrations of 12.5, 25, 62.5, and 125 μg/mL, making the final SiNP concentrations at 10, 20, 50, and 100 μg/mL. Double-distilled water and 0.8 mL of PBS were added to 0.2 mL of diluted RBC suspensions as the positive and negative control groups, respectively. All samples were gently vortexed and prepared in triplicate. The mixtures were incubated at 37 °C for 30 min and 2 h. After incubation, all samples were centrifuged at 3500 rpm for 5 min. One hundred microliters of supernatant from the tube was transferred to a 96-well plate, and the absorbance of hemoglobin was measured by a microplate reader (Tecan, Australia) at 577 nm. The percent hemolysis of erythrocytes was calculated as percent hemolysis (%) = [(sample absorbance − negative control)/(positive control − negative control)]*100 %.

### Erythrocyte Morphology Assessment

An amount of 0.2 mL of diluted RBC suspensions was incubated at 37 °C for 2 h with amorphous SiNPs at the final concentrations of 0, 20, 50, and 100 μg/mL. After incubation, the RBC suspensions were dropped to a slide glass to make blood smear and fixed with methyl alcohol. The slides were stained with Giemsa dye (KeyGEN Biotech, China) according to the standard techniques and observed under the optical microscope (Olympus X71-F22PH, Japan). The magnification is ×400.

### Erythrocyte Membrane Isolation

Packed erythrocytes were obtained after the incubation with SiNPs at the final concentrations of 0, 10, 20, 50, and 100 μg/mL. Pre-cold lysis buffer (10 mM Tris-HCL, pH 7.4, and 0.03 mM PMSF) was added into the packed erythrocytes (volume ratio was about 40:1) and gently vortexed. The mixtures were incubated at 4 °C overnight. After incubation, the lytic erythrocytes were centrifuged at 9000 rpm for 20 min and the sediments were erythrocyte membranes. The membranes were washed three times with the pre-cold lysis buffer and stored in PBS solution (pH 7.4) at −20 °C until analysis.

### ESR Experiments

The hydroxyl radical (·OH) levels were calculated according to Fenton reaction. Five microliters of erythrocyte membrane solutions with different SiNP concentrations (0, 10, 20, 50, and 100 μg/mL) was added into the mixture of 5 μL FeSO_4_ · 7H_2_O (10 mM) and 5 μL spin-label DMPO (5,5-dimethyl-1-pyrroline *N*-oxide) (Sigma, USA). The same volume of PBS buffer (pH 7.4) was added into the blank tubes. After mixing well, 5 μL 1 % H_2_O_2_ was immediately added into both sample tubes and blank tubes and reacted for 2 min before measurement. DMPO–OH adduct was generated to detect ·OH levels. The mixtures were gently vortexed and carefully transferred to a VC-H075P plain-type hematocrit capillary tube (Terumo Corp. Tokyo, Japan) with an RUVF-203S glass fiber UV irradiator (Radical Research, Tokyo, Japan, 200 W Hg-Xe lamp). After irradiation, the spectrum of mixed solutions was measured by a JES TE-100 X-band ESR spectrometer (Jeol, Tokyo, Japan). The ESR spectra were recorded at 1-mW microwave power, 335.7-mT center field, ±l7.5-mT sweep width, and ±0.35-mT modulation width and controlled by a WIN-RAD ESR data analyzer (Radical Research, Tokyo, Japan) via detection of the peak heights of the spectra. The ·OH elimination rates were calculated as ·OH elimination rates (%) = [(peak heights of blank tubes − peak heights of sample tubes)/peak heights of blank tubes]*100 %.

### Oxidative Damage Measurement

An amount of 0.2 mL of diluted RBC suspensions was incubated at 37 °C for 2 h with amorphous SiNPs at the final concentrations of 0, 10, 20, 50, and 100 μg/mL. The mixtures were centrifuged at 3500 rpm for 5 min, and the supernatants were moved out to get the packed erythrocytes. Pre-cold double-distilled water was added to dissolve the erythrocytes, and RBC extraction liquid was obtained. The malondialdehyde (MDA) contents of erythrocytes were measured with thiobarbituric acid method and the superoxide dismutase (SOD) levels were quantified with hydroxylamine method according to procedures described in the commercially available kit (Jiancheng, China). The absorbance value of MDA was detected by a UV-vis spectrophotometer (Shimadzu, Japan) at 532 nm, and the SOD activity was determined at 550 nm. The hemoglobin concentrations in erythrocytes were measured by performing the bicinchoninic acid (BCA) protein assay (Pierce, USA).

### ATPase Activity Assessment

Ten microliters of sodium citrate-stabilized whole blood was diluted to 90 μL 0.9 % normal saline and briefly vortexed. The RBC suspensions were incubated at 37 °C for 2 h with amorphous SiNPs at the final concentrations of 0, 10, 20, 50, and 100 μg/mL. After incubation, the mixtures were centrifuged at 3500 rpm for 5 min and the supernatants were moved out to get the packed erythrocytes. Five microliters of washed erythrocyte was mixed to 245 μL pre-cold double-distilled water to be fully dissolved. Na^+^-K^+^ ATPase activity and Ca^2+^-Mg^2+^ ATPase activity were determined with molybdenum blue spectrophotometric method by detecting the phosphorus (Pi) content produced by transformation of adenosine triphosphate (ATP) to adenosine diphosphate (ADP). Corresponding assay kits were used (Jiancheng, China). The absorbance value was detected by a UV-vis spectrophotometer (Shimadzu, Japan) at 636 nm. The hemoglobin concentrations in erythrocytes were measured by performing the BCA protein assay (Pierce, USA).

### Hemolysis Assay Added with NAC and ADP

An amount of 0.2 mL of diluted RBC suspensions was respectively incubated with 0.8 mL of NAC dissolved in PBS at the final NAC concentrations of 0, 1, 2, 5, and 10 mM/L at 37 °C for 2 h, and the percent hemolysis was measured. An amount of 0.2 mL of diluted RBC suspensions was respectively pre-incubated with 0.4 mL of NAC dissolved in PBS at the final NAC concentrations of 0, 1, 2, 5, and 10 mM/L at 37 °C for 1 h. The mixtures were added with 0.4 mL of amorphous SiNP suspensions at the final concentration of 20 μg/mL and incubated at 37 °C for 2 h. An amount of 0.2 mL of diluted RBC suspensions was incubated with 0.8 mL of amorphous SiNP suspensions at the final concentration of 100 μg/mL; meanwhile, ADP solutions dissolved in 0.9 % normal saline were added into the suspensions at the final ADP concentrations of 0, 2, 5, 10, and 20 μM/L. The mixtures were incubated at 37 °C for 2 h. The percent hemolysis was measured following the same procedures described in previous methods.

### Statistical Analysis

Data were expressed as mean ± standard deviation (S.D.), and the significance of statistical comparisons among groups was determined by one-way analysis of variance (ANOVA) using SPSS Statistics 21. The normal distribution of our data was tested before analysis. Differences were considered significant at *p < 0.05.

## Results

### Characterization of Amorphous Silica Nanoparticles

**Fig. 1:**
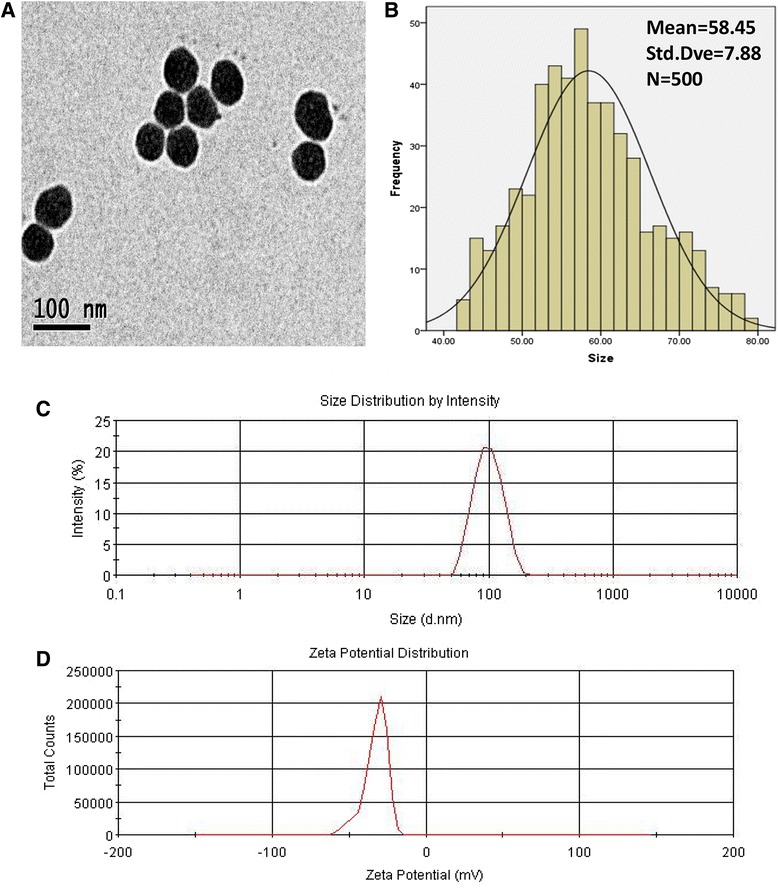
Characterization of amorphous SiNPs. **a** TEM images showed that SiNPs dispersed in PBS appeared to have a spherical shape and good monodispersity (*scale bar* 100 nm). **b** The size distribution of SiNPs showed approximately normal distribution. **c** Hydrodynamic size distribution and **d** zeta potential distribution of SiNPs suspended in PBS

TEM images showed that the amorphous SiNPs appeared to have a spherical shape (Fig. 1a) and the average size calculated by Image J software was 58.45 ± 7.88 nm, showing approximately normal distribution (Fig. 1b). The average value of the zeta potential was −35.3 mV, and the hydrodynamic sizes of SiNPs were about 95.31 nm, suggesting a good monodispersity in medium (Fig. 1c, d). The purity of SiNPs was confirmed higher than 99.9 % [26]. The results of the endotoxin in SiNP suspensions showed negative.

### Hemolytic Effects of SiNPs in Erythrocytes

**Fig. 2:**
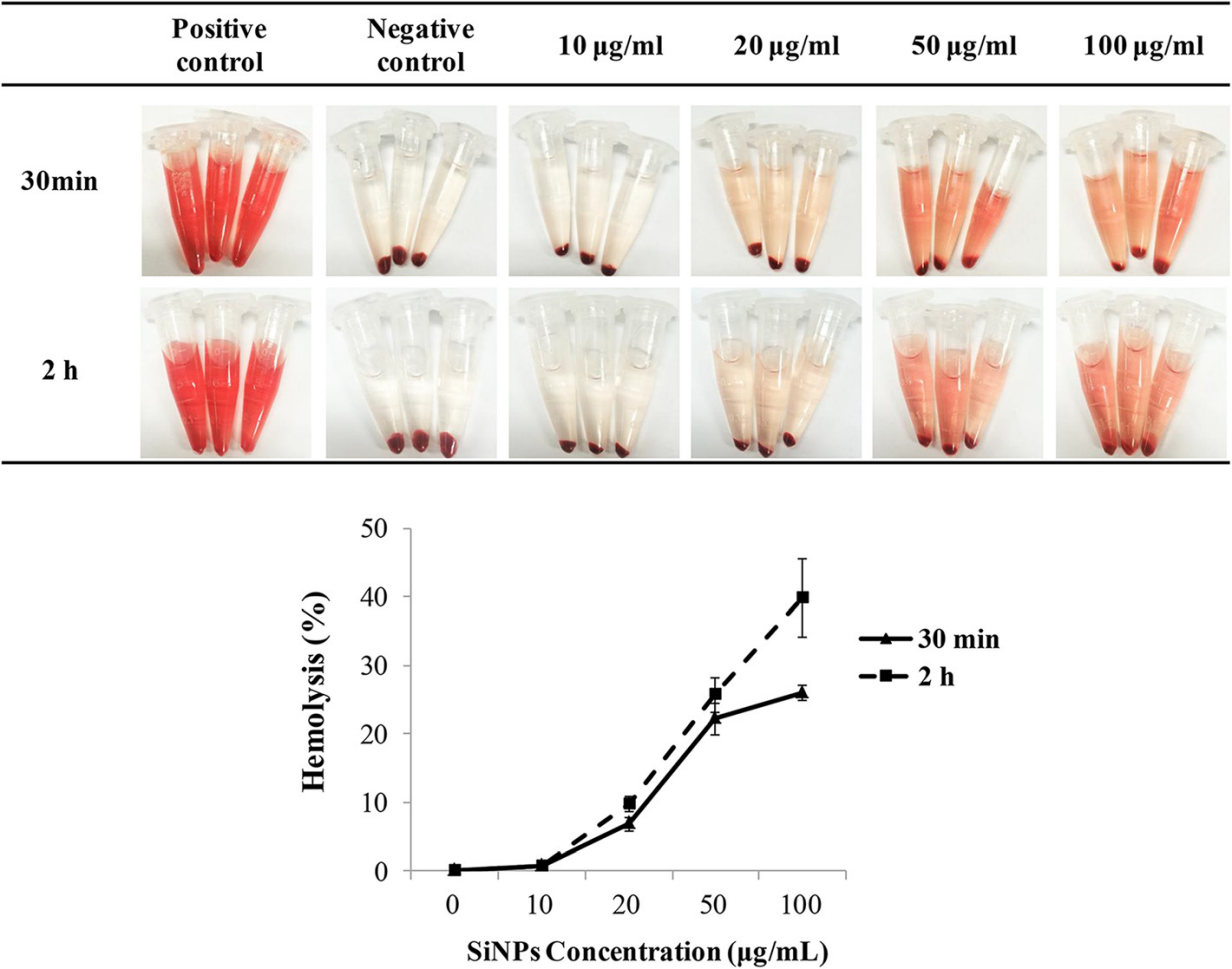
Photographs and percent hemolysis of human erythrocytes. Human erythrocytes were incubated with amorphous SiNPs at different concentrations ( 0, 10, 20, 50, and 100 μg/mL) for different exposure times (30 min and 2 h). Double-distilled water (+) and PBS (−) were used as positive and negative controls, respectively. Percent hemolysis (%) = [(sample absorbance − negative control)/(positive control − negative control)]*100 %. Data are expressed as mean ± S.D., *p < 0.05 vs the control group

As seen in Fig. 2, the color of supernatants is gradually deepening with the increased concentrations of SiNPs both in the 30-min and 2-h groups, suggesting more levels of hemoglobin were released from the ruptured erythrocytes induced by a higher dose of SiNPs. To quantify the hemolytic effects of SiNPs in human erythrocytes, percent hemolysis was calculated. The percent hemolysis of erythrocytes induced by SiNPs showed no significant changes at the concentration of 10 μg/mL at 30 min and 2 h, but at 20 μg/mL, the percent hemolysis in two time points showed significant increases in a dose-dependent manner (*p* < 0.05). Particularly mentioned, in the 100 μg/mL SiNP-treated groups, the percent hemolysis showed significant elevation after a 2-h incubation compared to the 30-min incubation (*p* < 0.05). Our results suggested SiNPs induced the hemolytic effects in human erythrocytes in a dose-dependent manner and the hemolysis increased remarkably after longer time exposure.

### Effects of SiNPs on Erythrocyte Morphology

**Fig. 3:**
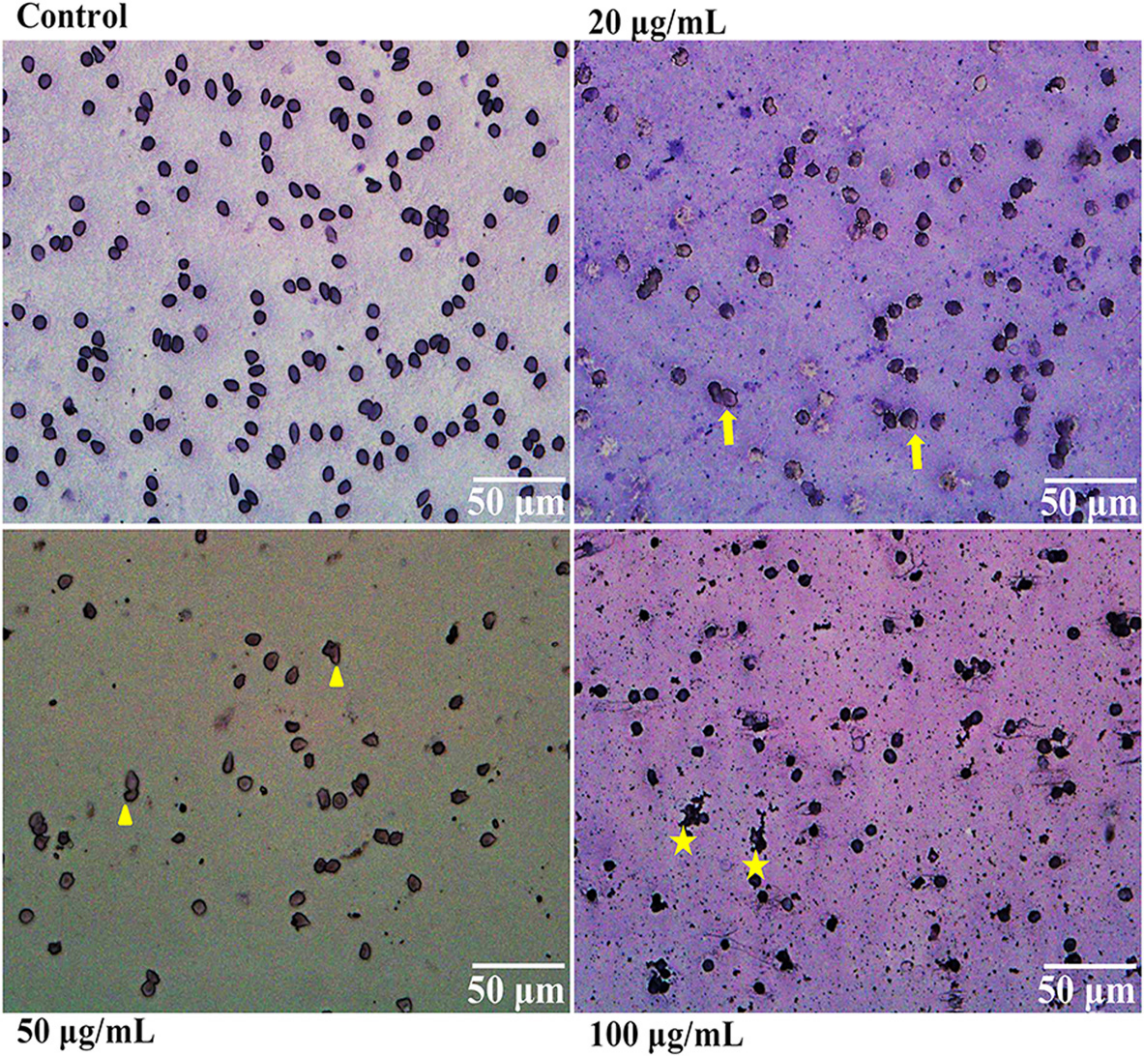
Giemsa staining of human erythrocyte smears. Human erythrocytes were incubated with amorphous SiNPs for 2 h at different concentrations ( 0, 10, 20, 50, and 100 μg/mL). *Arrows* represented the swelling erythrocytes; *triangles* represented the deformed erythrocytes; *five-point stars* represented the erythrocyte membrane fragments. The magnification was ×400. *Scale bars* are 50 μm

Figure 3 shows erythrocyte morphology at ×400 magnification after SiNP incubation with different concentrations (0, 20, 50, and 100 μg/mL). The erythrocytes in the control group displayed a typical concave disk and relatively uniform shape, size, and color. In the group of SiNPs, erythrocytes appeared swollen (arrows) and to have an irregular shape (triangles) and even membrane fragments (five-point stars). The density of erythrocytes in views was gradually reduced, which might explain that erythrocytes were disrupted by SiNPs.

### Effects of SiNPs on ·OH Elimination

**Fig. 4:**
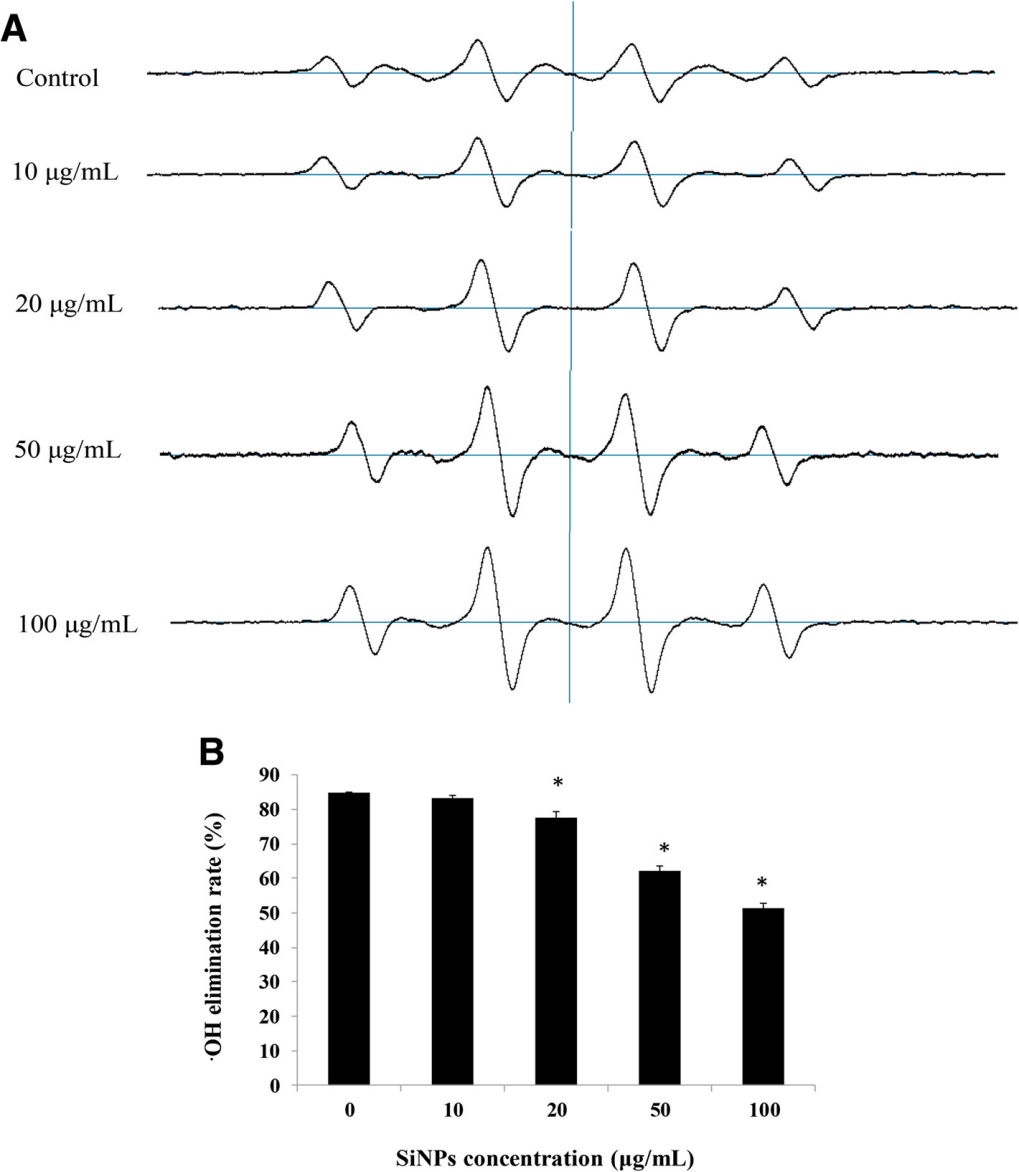
Effects of amorphous SiNPs on hydroxyl radical (·OH) elimination. **a** ESR spectra of ·OH in human erythrocyte membranes incubated with SiNPs for 2 h at different concentrations (0, 10, 20, 50, and 100 μg/mL). **b** ·OH elimination rates calculated as [(peak heights of blank tubes − peak heights of samples)/peak heights of blank tubes]*100 %. Data are expressed as mean ± S.D., *p < 0.05 vs the control group

To detect the ROS generation induced by SiNPs in human erythrocytes, ·OH levels of erythrocyte membranes were measured by ESR. Under the physiological conditions, the balance between the generation and elimination of ·OH can be maintained by the oxidation-antioxidation systems. As seen in Fig. 4, the spectrum peaks showed a gradually increased trend, indicating high levels of ·OH accumulation, giving evidences that the ability to eliminate ·OH in erythrocytes was inhibited. The elimination rates of ·OH were calculated to quantify the low activities of the antioxidation system. As it turned out from 20 μg/mL, SiNPs induced a dose-dependent decrease in the ·OH elimination rates and showed significant differences compared to the control group (*p* < 0.05).

### Effects of SiNPs on the MDA Content and SOD Activity

**Fig. 5:**
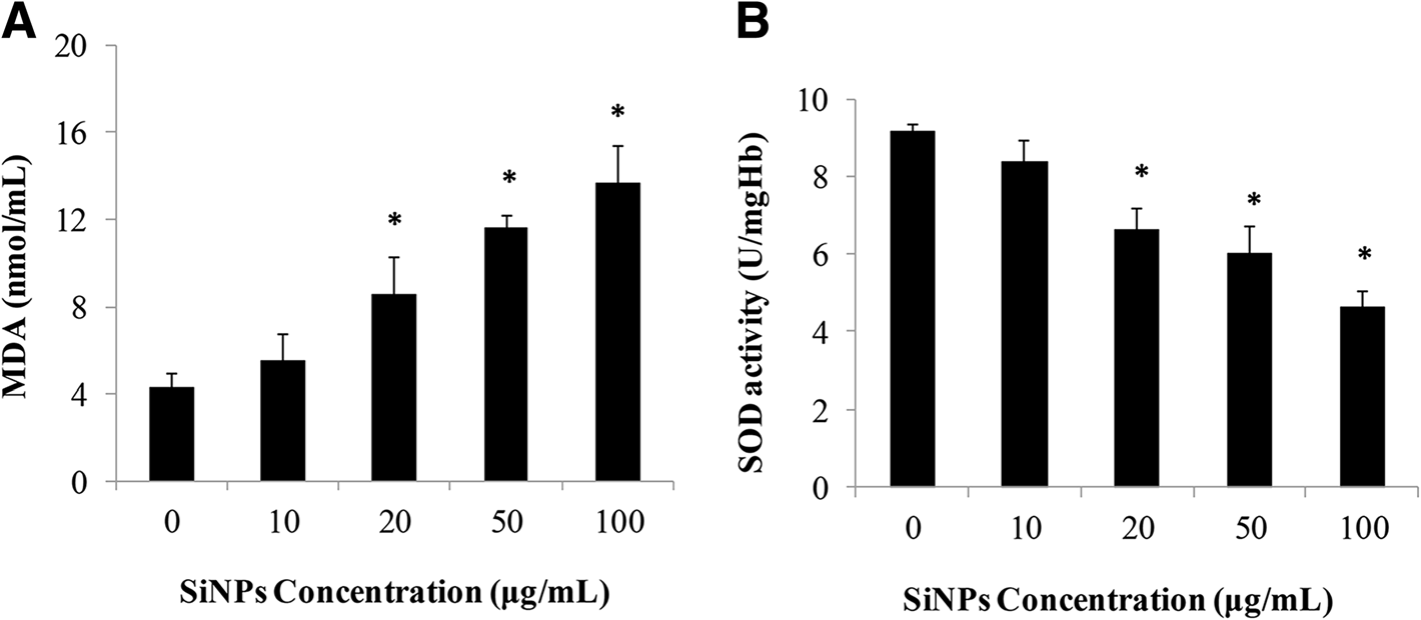
Effects of amorphous SiNPs on oxidative damage. Human erythrocytes were incubated with SiNPs for 2 h at different concentrations (0, 10, 20, 50, and 100 μg/mL). **a** Malondialdehyde (*MDA*) content. **b** Superoxide dismutase (*SOD*) activity. Data are expressed as mean ± S.D., *p < 0.05 vs the control group

To get further knowledge about the mechanisms of erythrocyte membrane disruption, we measured the MDA content and SOD activity after SiNP incubation at different concentrations. As shown in Fig. 5a, b, at the low concentration of SiNPs (10 μg/mL), there were no significant differences in the MDA content and SOD activity between the control and SiNP-treated groups, which was quite consistent with the effect of SiNPs on erythrocyte hemolysis. Figure 5a shows that SiNPs induced a dose-dependent increase in MDA concentrations and the effects were significant in the groups of 20, 50, and 100 μg/mL (*p* < 0.05). Contrary to the MDA levels, the SOD activity showed a decreased trend with the increased dose of SiNPs and the significances in comparison with the control group were discovered from 20 μg/mL to the highest levels (100 μg/mL) (*p* < 0.05) (Fig. 5b). The changes on the MDA content and SOD activity suggested amorphous SiNPs could cause oxidative damage in human erythrocytes, which was mainly responsible for erythrocytes hemolysis.

### Effects of SiNPs on ATPase Activities

**Fig. 6:**
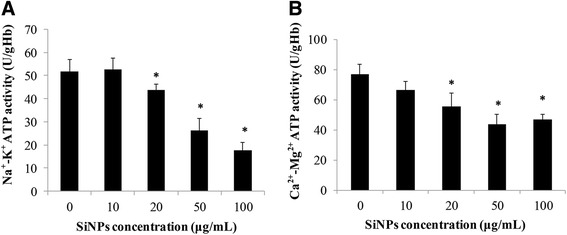
Effects of amorphous SiNPs on ATPase activities. Human erythrocytes were incubated with SiNPs for 2 h at different concentrations (0, 10, 20, 50, and 100 μg/mL). **a** Na^+^-K^+^ ATPase activity. **b** Ca^2+^-Mg^2+^ ATPase activity. Data are expressed as mean ± S.D., *p < 0.05 vs the control group

To measure the effects of SiNPs on energy metabolism of erythrocyte membrane, Na^+^-K^+^ ATPase and Ca^2+^-Mg^2+^ ATPase activity were detected. Data from Fig. 6a reveals a remarkable decrease in Na^+^-K^+^ ATPase activity in a dose-dependent manner, and it was down-regulated significantly in the groups of 20, 50, and 100 μg/mL compared to the control group (*p* < 0.05). As shown in Fig. 6b, Ca^2+^-Mg^2+^ ATPase activity was decreased in 10 μg/mL, but not significantly. At 20 to 100 μg/mL, there were significant differences in Ca^2+^-Mg^2+^ ATPase activity between the SiNP-treated groups and the control group (*p* < 0.05). Our results revealed that SiNPs could damage erythrocyte membranes by disturbing the ATPase activities and inducing the ATP metabolism disorder.

### Protection of NAC and ADP on Erythrocytes Hemolysis

**Fig. 7:**
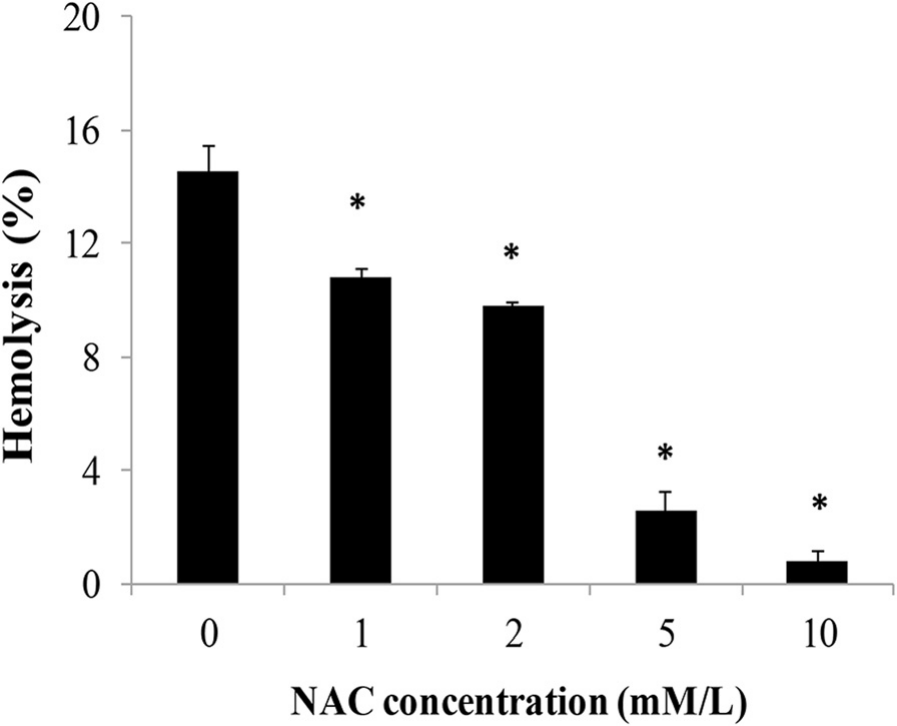
Effects of *N*-acetyl-cysteine (*NAC*) on percent hemolysis. Human erythrocytes with the pretreatment of NAC at different concentrations (0, 1, 2, 5, and 10 mM/L) were incubated with amorphous SiNPs of 20 μg/mL for 2 h. Data are expressed as mean ± S.D., *p < 0.05 vs the control group

We have noticed that ROS accumulation and oxidative damage might get involved in the erythrocyte membrane disruption; therefore, we chose NAC, which is an oxygen inhibitor to testify that oxidative stress contributed to hemolysis induced by SiNPs. Table 1 shows that there was no significant difference in percent hemolysis between groups only incubated with NAC (0, 1, 2, 5, and 10 mM/L), suggesting that NAC itself was incapable of inducing hemolysis. Figure 7 indicates that in the SiNP-treated groups with NAC, percent hemolysis showed an obvious dose-dependent decrease, and it was noteworthy that all groups in the presence of NAC showed significant differences compared to the control group in the absence of NAC (*p* < 0.05). Our data demonstrated that NAC successfully reduced the oxidative damage and played a protective role in the hemolytic effects of SiNPs in human erythrocytes.

**Table 1:** Percent hemolysis of human erythrocytes after NAC pretreatment

Group	Concentration (mM/L)	Percent hemolysis (%)
Control	0	0.000 ± 0.047
NAC	1	0.179 ± 0.111
	2	−0.099 ± 0.121
	5	−0.040 ± 0.090
	10	−0.051 ± 0.018

Data are expressed as mean ± S.D.

*p < 0.05 vs the control group

**Fig. 8:**
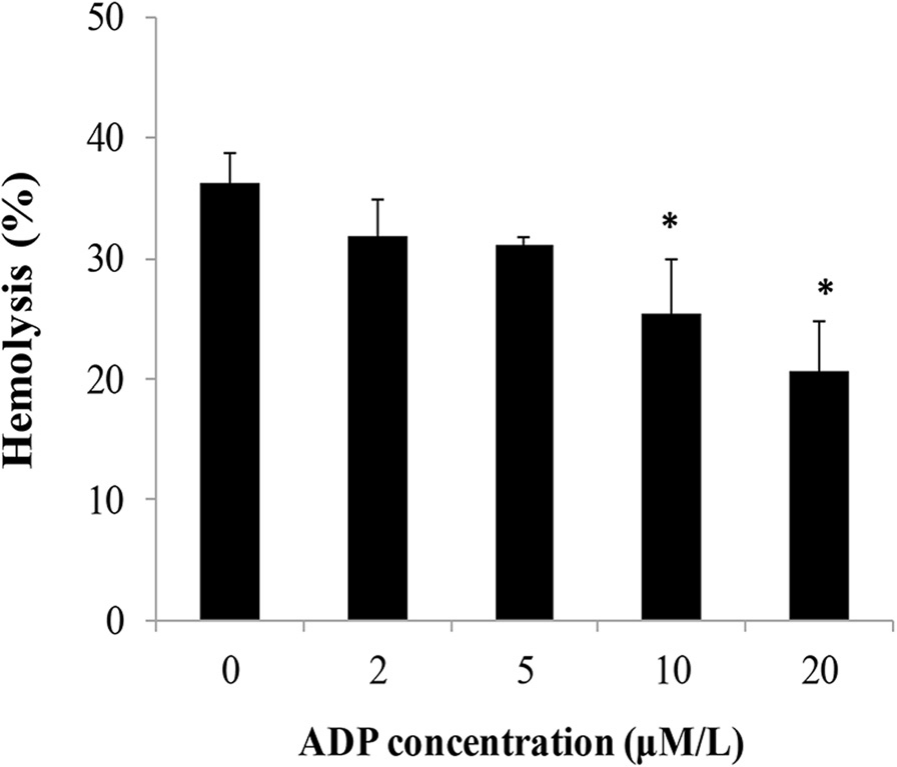
Effects of adenosine diphosphate (*ADP*) on percent hemolysis. Human erythrocytes with treatment of ADP at different concentrations (0, 2, 5, 10, and 20 μM/L) were incubated with amorphous SiNPs of 100 μg/mL for 2 h. Data are expressed as mean ± S.D., *p < 0.05 vs the control group

Figure 8 shows the effects on percent hemolysis of human erythrocytes after SiNP incubation with ADP. From our results, the hemolysis was significantly decreased at the ADP concentrations of 10 and 20 μM/L (*p* < 0.05), which suggested that as a synthesis source of ATP, ADP was conducive to reduce the hemolytic effects of SiNPs. In other words, ATP shortage caused by ATPase activity damage is a reasonable explanation for hemolysis induced by SiNPs.

## Discussion

The bloodstream exposure to SiNPs through environmental and iatrogenic routes arouses great concerns on the biocompatibility and hemostability of SiNPs. In a previous study, we have noticed that SiNPs could induce hemolytic anemia in vivo (unpublished data), and to further clarify the hemolytic potentials of SiNPs on erythrocytes and the propensity to induce RBC membrane deformation or morphological alteration, the hemolysis assay in vitro was performed. PBS which is protein-free and isotonic to the blood was selected as the culture solution to strip out the impact of proteins and ions on SiNP characterization.

As mentioned above, erythrocyte species probably acted as an important impact factor for hemolytic activity of nanoparticles. The difference in amplitude of the hemolytic effects was explained due to the different susceptibility of nanoparticles interacting with erythrocyte membrane of various species [16]. In the present study, amorphous SiNPs ruptured human erythrocytes and led to significant hemolysis (Fig. 2), indicating human erythrocyte membrane was sensitive to amorphous SiNPs. It has been reported that amorphous SiNPs induced dose-dependent hemolysis in mice [18]; however, we testified the hemolytic effects of SiNPs on human RBCs, providing new insight for safety evaluation of SiNPs. In order to find out if time extension affected the hemolysis in erythrocytes, we tested the percent hemolysis at different time points (30 min and 2 h) after SiNP exposure. It turned out that the hemolysis was enhanced as the exposure time elongated (Fig. 2).

Red cell membrane rupture or the matrix dissociation are the essential reasons for hemolysis to occur. In various cell lines, it was confirmed that amorphous SiNPs induced cytotoxicity via the disruption of cell membrane integrity and the collapse of the cytoskeleton [31–33]. Cell membrane permeability is usually measured by lactic dehydrogenase (LDH) activity in cell medium, which is an effective indictor for irreversible cell death. According to our previous study, LDH activity showed a dose-dependent increase both in human endothelial cells [32] and the erythrocyte supernatants after amorphous SiNP incubation (unpublished observations). These results showed evidences that SiNPs exerted hemolytic effects via increasing the erythrocyte membrane permeability and promoting red cell rupture. The morphological observation of erythrocytes via Giemsa staining found that amorphous SiNPs could cause erythrocyte deformation, cell swelling, and cytomembrane damage or even rupture (Fig. 3).

To further investigate the potential mechanisms of erythrocyte membrane rupture caused by SiNPs, the oxidative damage in human erythrocytes was measured. Oxidative stress is a consequence of imbalances in oxidation and antioxidation. Oxidative damage was considered as a common toxic mechanism of SiNPs for decreasing cell viability and breaking membrane integrity [34]. Owing to specific physicochemical properties, the SiNPs did not only generate spontaneous ROS at the surface but also triggered free oxygen radicals on cells [24]. The persistent accumulation of ROS induced by SiNPs could result in peroxidation of cell components like proteins, lipids, and DNA and decrease cellular antioxidant activity [23]. Hydroxyl radical (·OH) is one kind of important and highly active ROS, which was generally accepted as the main cause of biological damage [35]. It was demonstrated that both quartz and vitreous silica stably induced surface radicals and sustained release of ·OH [36]. Amorphous SiNPs up-regulated heme oxygenase-1 (HO-1) mRNA expression in endothelial cells, and ·OH production induced by SiNPs was considered as the primary cause for the decreased cell viability [37]. The decreased ·OH elimination rates in our study indicated that SiNPs inhibited the antioxidant activity to facilitate ROS accumulation and ·OH played an active role in hemolysis (Fig. 4). MDA is a final product of lipid peroxidation, generated from the peroxidation of polyunsaturated fatty acid (PUFA) attacked by the free radicals [38]. Moreover, the more free radicals were produced, the more serious lipid peroxidation and oxidative damage in cell functions were induced. In general, MDA is treated as a reliable indicator to measure the oxidative damage [38]. It was reported that lipid peroxide was treated as key process to erythrocyte membrane disruption after exposure to DEP [16] and incubation of mouse erythrocytes with SiNPs (50 nm) caused significant increase in the MDA content [18], which were fairly consistent with our results of the MDA content (Fig. 5a). These findings suggested that erythrocyte membrane was a direct target that SiNPs interacted with and the lipid peroxidation of membrane was a potential mechanism of membrane damage. SOD is a kind of antioxidant enzyme which plays a vital role in maintaining oxidative homeostasis. Previously, we have reported that amorphous SiNPs induced a significant decrease in SOD activity and glutathione peroxidase (GSH-PX) activity in human umbilical vein endothelial cells (HUVECs), leading to endothelial injury, cell autophagy, and apoptosis [23–25]. Herein, the decreased SOD activity (Fig. 5b) demonstrated the insufficient antioxidant ability of erythrocytes to resist the oxidation induced by SiNPs, resulting in erythrocyte damage and rupture. To further elucidate the relationship between ROS and hemolysis in erythrocytes, *N*-acetyl-cysteine (NAC) which is a regular scavenger of ROS was added to alter the percent hemolysis of SiNPs. The ROS toxicity can be eliminated via combination of the ROS electrophilic groups with the NAC sulfhydryl group. Moreover, NAC is a precursor of glutathione, playing important roles in antioxidation owing to its good permeability to cell membrane [39, 40]. The resistance of NAC in oxidative damage towards nanomaterials has been well documented, and cell multinucleation and autophagy induced by amorphous SiNPs were remarkably alleviated by NAC [25, 41]. Accordingly, the percent hemolysis showed a dose-dependent decrease after NAC treatment (Fig. 7), suggesting that ROS generation was closely related to the hemolysis in erythrocytes. Our data suggested that amorphous SiNPs induced a dose-dependent ROS production, leading to the lipid peroxidation and oxidative damage of erythrocyte membrane, consequently causing hemolysis.

Endocytosis was considered as the common way involved in the interaction of SiNPs with the red cell membrane [10, 42], and the occurrence of endocytosis relied heavily on the released energy on the surface of the membrane and the deformability of the membrane [43]. To measure the energy metabolism of erythrocytes in our study, Na^+^-K^+^ ATPase and Ca^2+^-Mg^2+^ ATPase activities were detected. Na^+^-K^+^ ATPase is a membranous protein, which is capable of providing energy released from ATP hydrolysis and maintaining the ion concentration gradients across the membrane of low Na^+^ levels inside and low K^+^ levels outside the cells [44, 45]. The insufficiency and exhaust of ATP was a potential toxic mechanism of erythrocyte caducity by inducing abnormal ion transport, energy metabolism suffocation, and declined erythrocyte deformability [46]. It was demonstrated that zinc oxide nanoparticles (ZnO) decreased the Na^+^-K^+^ ATPase activity in freshwater fish leading to epithelial permeability increase and structure remodeling [47]. In addition, silver nanoparticles (AgNPs) inhibited the Na^+^-K^+^ ATPase and erythrocyte acetylcholinestrase (AChE) activities, resulting in improper ion regulation and energy imbalance [48]. The decreased Na^+^-K^+^ ATPase activity (Fig. 6a) suggested that amorphous SiNPs could reduce the capacity of energy generation in human erythrocytes, followed by red cell membrane dysfunction and disruption. Membrane calcium ion (Ca^2+^) ATPase is a high-affinity Ca^2+^ pump which takes charge of the regulation of intracellular Ca^2+^ levels and releases energy from driving Ca^2+^ out of cells [49]. Ca^2+^ participates in the regulation of various vital functions in cells, such as cell signal transduction, differentiation, and metabolism [50]. Wu et al. found that TiO_2_ nanoparticle could efficiently inhibit the cell proliferation in human lens epithelial cells via disruption of calcium homeostasis of elevating intracellular Ca^2+^ and inhibiting Ca^2+^ ATPase activities [51]. The exposure of rat erythrocytes to amorphous SiNPs caused remarkable increase in intracellular Ca^2+^ levels and Annexin V expression which is a Ca^2+^-dependent binding protein, leading to apoptosis of erythrocytes [18]. Ca^2+^ ATPase was confirmed to be susceptible to oxidative damage because of the sulfhydryl structure, and thus, Ca^2+^ ATPase activities were sensitive to the oxidative homeostasis alteration [52]. In the present study, SiNPs inhibited the Ca^2+^-Mg^2+^ ATPase activity in human erythrocytes (Fig. 6b), possibly because of the high intracellular Ca^2+^ levels and interference effects of oxidative stress. ADP is a source for synthesis of ATP, and the decreased percent hemolysis of SiNPs in erythrocytes incubated with ADP (Fig. 8) was persuasive that ATP was necessary for maintaining membrane integrity. Combined with the damage of Na^+^-K^+^ ATPase and Ca^2+^-Mg^2+^ ATPase activities and the protective effects of ADP on hemolysis, we believed that energy metabolism imbalance and ion channel dysfunction of erythrocyte membrane were largely accountable for hemolysis induced by amorphous SiNPs.

## Conclusions

It was concluded that amorphous SiNPs caused dose-dependent hemolytic effects and remarkably altered the shape and deformability of erythrocytes. The abilities of ·OH elimination were inhibited by SiNPs to facilitate ROS accumulation in erythrocytes, and ·OH played an active role in hemolysis. SiNPs induced a dose-dependent ROS production, leading to oxidative damage of erythrocyte membrane by increasing the lipid peroxidation and decreasing the antioxidant ability, consequently causing hemolysis. ATPase activities were inhibited, and the energy metabolism disorder of membrane contributed to the hemolytic effects of SiNPs in human erythrocytes. Our findings provided a new vision to lucubrate the hematotoxicity of amorphous SiNPs, but the mechanism in depth of hemolysis and the risk assessment and safety evaluation of SiNPs in biomedical applications are still needed.

## Abbreviations

AChE: acetylcholinestrase
ADP: adenosine diphosphate
AgNPs: silver nanoparticles
ANOVA: analysis of variance
ATP: adenosine triphosphate
BCA: bicinchoninic acid
Ca^2+^: calcium ions
DEP: diesel exhaust particles
DMPO: 5,5-dimethyl-1-pyrroline *N*-oxide
ESR: electron spin resonance
GSH-PX: glutathione peroxidase
LDH: lactic dehydrogenase
MDA: malondialdehyde
MSN: mesoporous silica nanoparticles
NAC: *N*-acetyl-cysteine
·OH: hydroxyl radical
PBS: phosphate-buffered saline
RBC: red blood cell
ROS: reactive oxygen
SiNPs: silica nanoparticles
SOD: superoxide dismutase
